# Supplementary material for: The Evolution and Characterization of the RNA Interference Pathways in Lophotrochozoa
Source: Genome Biol Evol. 2024 May 7;16(5):evae098. doi: 10.1093/gbe/evae098 (PMC11114477; doi:10.1093/gbe/evae098)
Supplement: evae098_Supplementary_Data [file evae098_supplementary_data.zip › Supplementary_materials.zip!/selected_miRNAs/NoGe_selected_miRNAs/known_miRNA_families_pdf_files/NMRB01000654.1_Notospermus_geniculatus_strain_Ushimado_scaffold654,_whole_genome_shotgun_sequence_17940.pdf]

| | | |
|---|---|---|
| Provisional ID | : | NMRB01000654.1_Notospermus_geniculatus_strain_Ushimado_scaffold654,_whole_genome_shotgun_sequence_17940 |
| Score total | : | 29978.2 |
| Score for star read(s) | : | 3.9 |
| Score for read counts | : | 29969.7 |
| Score for mfe | : | 1.6 |
| Score for randfold | : | 0 |
| Score for cons. seed | : | 3 |
| Total read count | : | 58796 |
| Mature read count | : | 57058 |
| Loop read count | : | 0 |
| Star read count | : | 1738 |

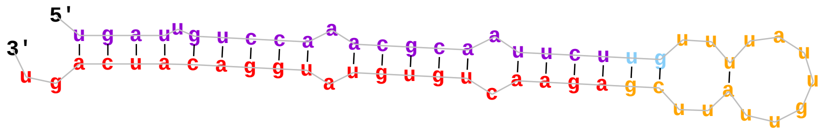


Genome coordinates: NMRB01000654.1 34703-34763

pre-miRNA sequence:
UGAUUGUCCAAACGCAAUUCUUGAUUGUCCAAACGCAAUUCUUGUUUUAUUGUUAUUCGAGAACUGUGUAUGGACAUCAGU

Mature sequence: AGAACUGUGUAUGGACAUCAGU

Aligned mature sequence of the *N. geniculatus* pre-miRNA with the top 5 hits from the BLAST search against the pre-miRNA mirGeneDB set:

```
                            ** ******* *********
Nge_Mir-219                 agaacuguguauggacaucagu
Tca-Mir-219_3p              aggacugugugugacaucauc
Lpo-Mir-219-P22_3p          agaacugugugugacaucagu
Lan-Mir-219_3p              agaacuguguauggacaucagu
Esc-Mir-219_3p              agaacuguguuuggacaucagu
Pmi-Mir-219_3p              agaacuguguauggacaucagu
```
